# Immune cells: the key mediator between the gut microbiota and osteoporosis

**DOI:** 10.3389/fimmu.2025.1680021

**Published:** 2025-10-02

**Authors:** Tianyi Ma, Tiantian Zhang, Chengqi Peng, Ke Liu, Yixiao Xiong, Keru Chen, Nazi Peng, Zhentao Wei, Jianjun Kuang, Liang Ou

**Affiliations:** ^1^ Hunan University of Chinese Medicine, Changsha, China; ^2^ Hunan Academy of Chinese Medicine, Changsha, China; ^3^ Affiliated Hospital of Hunan Academy of Traditional Chinese Medicine, Changsha, China

**Keywords:** gut microbiota, intestinal flora, immune cells, osteoporosis, osteolmmunology

## Abstract

As the body’s largest immunological interface, the intestine harbors a complex ecosystem of gut microbiota (GM) that orchestrates mucosal immune maturation while sustaining local immunological equilibrium. Emerging evidence reveals the gut’s influence on skeletal homeostasis via neuro-immune-endocrine pathways—termed the gut-bone axis—though its mechanistic intricacies remain incompletely defined. Since the concept of osteoimmunology was proposed in 2000 by Arron & Choi, immune-skeletal interactions have garnered significant research traction. Immune cells primarily contribute to the maintenance of bone homeostasis through the release of pro- and anti-inflammatory factors. Consequently, the immune system represents a crucial intermediary in understanding the relationship between GM and metabolic bone diseases. This review synthesizes the interrelationships among gut microbiota, immune cells, and osteoporosis, and elucidates how GM modulate bone metabolism in osteoporosis through this critical intermediary. Furthermore, building upon the microbiome–immune–bone axis, we highlight several emerging microbiota-targeted interventions—such as probiotics, prebiotics, dietary modifications, fecal microbiota transplantation, and engineered microbes—and evaluate their clinical translational potential, with the aim of advancing diagnostic and therapeutic strategies for metabolic bone disorders.

## 1 Introduction

Osteoporosis (OP), a common systemic metabolic bone disease, is characterized by low bone mass and structural deterioration of bone tissue leading to bone fragility and an increased risk for fractures (1). Globally, over 200 million people suffer from OP (2). And it is estimated that OP will be a significant healthcare burden in the future due to the increased incidence of the disease with age and female gender. Currently, the underlying molecular mechanisms of OP pathogenesis continue to be investigated. With the in-depth study of gut microbiome (GM), some studies have demonstrated decreased bacterial richness and diversity in patient with postmenopausal osteoporosis (PMOP) (3).

The GM is now viewed as a tissue that interacts bidirectionally with the gastrointestinal, immune, endocrine and nervous systems, affecting the cellular responses in numerous organs (4, 5). Accumulating evidence suggests that the GM plays an important role in the regulation of the bone homeostasis and serves as a contributing mechanism of OP (5). Current research reveals that bone homeostasis is linked to a healthy microbiome and that gut dysbiosis can exacerbate osteoclasts (OCs) activity and promote OP (6). The GM can affect bone metabolism through multiple pathways including the intestinal barrier, metabolic pathways, nutrient absorption such as calcium and phosphorus, the immune system, and hormonal environment (7). However, the mechanism by which intestinal flora regulate bone homeostasis remains to be elucidated.

Meanwhile, as the largest immune organ in the human body, the GM is where most lymphocytes interact with other immune factors. The GM could regulate the maturation of the mucosal immune system, whereas the pathogenic microbiome causes immune dysfunction leading to disease development. In recent years, a growing number of studies have reported a close relationship among immune cells, GM and OP. And some studies indicated that intestinal flora regulates immunity and affects bone metabolism. Here, we summarize the relationship between immune cells and GM and OP, and show how the GM regulates OP through immune cells.

## 2 Gut microbiota and osteoporosis

The human gastrointestinal tract contains more than 10 trillion bacteria. As a result, the GM is also known as the second largest human genome. Recently, accumulating research confirmed that the diverse community of microorganisms has been recognized as a profound role in maintaining the bone homeostasis (8). It was found that the bacterial composition and diversity are altered in patients with OP compared to normal people, supporting the view that the pathological process of OP is affected by the GM (9). Herein, we attempted to explore the intimate connection between the GM and OP from the following three aspects.

### 2.1 The gut-bone axis

The intestinal microbiota is actively involved in many necessary physiological reactions. The gut interacts with most of the organs through neural, immune and endocrine pathways, a connection known as the gut-organ axis (10). Among them, the gut-bone axis mainly refers to the bidirectional relationship between the GM and bone tissue (11). Recently, the correlation between OP and intestinal microflora has been studied. Sjögren et al. conducted a study on germ-free (GF) mice and found GF mice exhibited decreased bone mineral density (BMD) compared to conventionally raised mice. Conversely, when the GM from conventionally raised mice was transferred to the GF mice, reduced bone density was reversed (12).

Their interplay tends to emphasize that the GM is a key regulator of bone health, suggesting its potential therapeutic targets for multiple bone disorders, especially for OP. A cross-sectional study reported that increased abundance of *Prevotellaceae* was positively associated with systemic inflammation and rheumatoid arthritis (RA) onset, suggesting that GM dysbiosis may contribute to bone loss via inflammatory mediators (13). As we know that intestinal inflammation can be accompanied by OP, but their relationship remains unclear. A new study induced food-allergic bowel disease using a non-IgE-mediated food-allergic enteropathy model of ovalbumin (OVA) 23–3 mice, found that abnormally activated OVA-specific Th2 cells in the mesenteric lymph nodes that overproduce IL-4 migrated to the bone marrow, trigger an inflammatory cascade that promotes bone damage (14). The findings reveal the mechanism by which the gut-bone axis plays an important role in bone loss induced by food-allergic bowel disease. Experimental evidence demonstrates that *Fusobacterium nucleatum* modulates M1 macrophage polarization via the AKT2 signaling pathway, exacerbating colonic inflammation and revealing a mechanism through which microbes influence bone metabolism via immune regulation (15). Subsequently, another research found that gut inflammation promotes OCs differentiation generation and bone loss by promoting cytokine changes, and upregulation of OCs precursor surface receptor MDL-1 expression (16). This work shows a relationship between gut inflammation and bone health, providing new evidence for gut-bone axis interactions. Xie Hui’s research team found that children’s intestinal flora, or the probiotic *Akkermansia muciniphila* in it, can promote bone formation and inhibit bone absorption by releasing extracellular vesicles (EVs) into bone tissue (17). It can reduce bone loss in PMOP mice. This study reveals a novel “gut-bone axis” regulatory model of bone metabolism mediated by gut bacteria functional EVs.

Altogether, the gut-bone axis offers a rationale for a potential therapeutic option for treating OP.

### 2.2 Gut microbiota and bone formation

OP occurs when there is an imbalance between bone formation and bone resorption during bone remodeling. A study employing integrated 16S rRNA gene sequencing and liquid chromatography-mass spectrometry metabolomic analysis revealed a positive correlation between *Bacteroides* abundance and BMD. The abundance of *Bacteroides* was significantly higher in the control group compared with the OP group, suggesting a potential regulatory role through the tryptophan-indole metabolic pathway. This finding indicates that GM dysbiosis may represent a significant risk factor for OP (18). Dysbiosis of intestinal microflora can lead to malabsorption of essential elements for bone growth, such as calcium, carbohydrates, B vitamins, and vitamin K, and to the production of metabolites that affect cellular signaling (19). Changes in the intestinal flora may thereby affect the calcium regulation and bone density. Studies have demonstrated that *Lactobacillus acidophilus*-fermented Astragalus polysaccharides alter the GM by increasing the relative abundance of specific beneficial bacteria and stimulating the production of key metabolites, thereby enhancing calcium absorption and ameliorating OP (20). Separately, sheep bone protein hydrolysate was shown to increase *Firmicutes* abundance, reduce *Proteobacteria* and *Verrucomicrobia*, promote short-chain fatty acids (SCFAs) production, improve calcium uptake, and ultimately restore BMD (21).

He W investigated the effect of the GM on calcium absorption in ovariectomized (OVX) rats, and found that specific species (e.g. *Acinetobacter* and *Propionibacterium*) of gut bacteria were associated with higher calcium absorption efficiency (22). There is a decrease in beneficial intestinal bacteria for postmenopausal women with OP, always accompanied with reduced levels of bone formation markers (23). Furthermore, studies have revealed that OVX mice exhibit a significant increase in the *Firmicutes/Bacteroidetes* ratio and elevated serum Lipopolysaccharide (LPS) levels, suggesting that estrogen deficiency may represent a key mechanism through which GM dysbiosis contributes to bone loss (24).

One of these studies reported that *Lactobacillus rhamnosus GG* regulating the intestinal microbiome and intestinal barrier through probiotics, stimulating the Th17/Treg balance in the intestine and bones, and promoting bone formation can improve OP caused by estrogen deficiency (25). Moreover, the combination of *Butyricicoccus pullicaecorum* and 3-hydroxyanthranilic acid (3-HAA) was shown to prevent PMOP by modulating the Th17/Treg immune balance, highlighting the potential of targeting the microbiota-immune axis for OP treatment (26).

Parathyroid hormone (PTH) is a critical regulator of skeletal development that promotes bone formation. Jau-Yi Li.et showed that the microbiota was required for PTH to stimulate bone formation and increase bone mass, and found butyrate, a metabolite responsible for gut-bone communication, plays in triggering regulatory pathways, which suggested that supplementing butyric acid to increase the number of regulatory T cells may be a new method to treat OP or enhance the anabolic activity of PTH (27). Supplementation with *Pseudobifidobacterium* CECT 7765 has been shown to upregulate the Wnt/β-catenin signaling pathway, resulting in increased levels of PTH, serum C-terminal telopeptide (CTX), and osteocalcin (28). In addition, two independent studies demonstrated that *Bacillus subtilis* enhances bone formation either by modulating bacterial communities to elevate PTH levels or by reducing TNF-α expression, thereby attenuating inflammatory conditions (29, 30).

Therefore, these studies highlight the potential of targeting GM to regulate bone formation in OP.

### 2.3 Gut microbiota and bone resorption

GM can influence bone resorption by modulating the immune and endocrine system, affecting inflammation, and producing metabolites that impact bone cell activity, including OCs.

Ohlsson investigated the role of GM in PMOP in mice (31). They found that the GM promoted bone loss by increasing the differentiation and activity of OCs, the cells responsible for bone resorption. Provided evidence that probiotic supplementation partially reverses the suppression of bone marrow T-regulatory cells caused by ovariectomy. The probiotic intake resulted in reduced levels of bone TNF-α and other pro-inflammatory cytokines in OVX mice, consistent with the regulation of anti-inflammatory T-cells (32, 33). Additionally, the researchers observed an increase in TGF-β1 expression, which is associated with enhanced T-regulatory cells (32). Li investigated the impact of GM dysbiosis on bone resorption in a mouse model of PMOP (34). They found that dysbiosis led to an altered GM profile, increased inflammation, and enhanced OCs activity, resulting in accelerated bone loss. The study highlighted the potential of targeting GM to modulate bone resorption in OP.

Wallimann found that GM and the metabolites they produce, primarily SCFAs, have been shown to affect almost all organs in the human body, including bones (35). SCFAs have shown a wide range of activities in positively affecting bone healing outcomes by acting directly on cell types involved in fracture healing, such as osteoblasts (OBs), OCs, chondrocytes, and fibroblasts, or indirectly on appropriate anti-inflammatory and immunomodulatory responses (35, 36). It is well documented that the GM can increase bone mass and improve OP by inhibiting OCs proliferation and differentiation, inducing apoptosis, reducing bone resorption, or promoting OBs proliferation and maturation. Experimental studies have demonstrated that quercetin supplementation ameliorates bone loss in OVX rats by enhancing intestinal barrier function through upregulation of tight junction proteins (ZO-1 and occludin), increasing SCFAs levels, and reducing pro-inflammatory mediators such as LPS, IL-1β, and TNF-α (37). These findings underscore the role of microbial-derived metabolites (e.g., SCFAs) in inhibiting bone resorption, highlighting a microbiota-metabolite-immune mechanism in OP intervention.

Peripheral serotonin (5-HT), produced by enterochromaffin (EC) cells in the gastrointestinal tract, promotes OCs differentiation and suppresses OBs activity. GM dysbiosis resulting from chronic ethanol abuse has been shown to increase 5-HT levels and exacerbate bone resorption (38). In contrast, centrally-derived 5-HT in the brain promotes bone formation by binding to Htr2c receptors on ventromedial hypothalamic neurons (39). Experimental supplementation with *Clostridium butyricum* and 25-hydroxyvitamin D_3_ (25-OH-D_3_) in broilers was found to increase 5-HT levels, decrease bone Gla protein (BGP) and peptide YY (PYY) levels, and improve bone metabolism. Metagenomic analysis of the cecum further revealed an increased abundance of *Alistipes* and significant alterations in metabolic pathways such as PI3K-AKT signaling and bile acid biosynthesis (40). Additionally, soybean germ extract and *Lactobacillus gasseri* exerted positive effects on skeletal health in OVX rats, significantly improving alkaline phosphatase (ALP) and bone resorption markers, while also elevating levels of estrogen, 5-HT, and norepinephrine (41).

**Figure 1:**
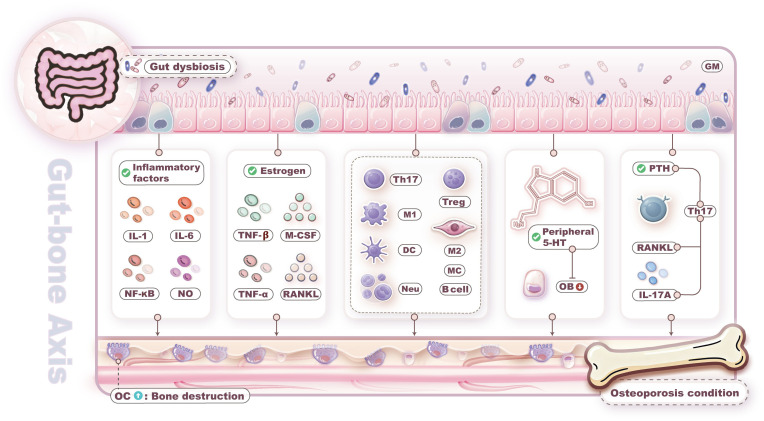
Gut microbiota (GM) dysbiosis leads to bone loss. Bone loss driven by GM dysbiosis through multiple mechanisms, including inflammatory factor release, metabolite alterations, immune dysregulation, intestinal barrier impairment, and endocrine hormone disruption.

In summary, bone loss within the gut-bone axis is primarily driven by GM dysbiosis through multiple mechanisms, including inflammatory factor release, metabolite alterations, immune dysregulation, intestinal barrier impairment, and endocrine hormone disruption. The mechanisms through which GM dysbiosis influences bone metabolism are illustrated in **Figure 1**.

## 3 Interaction between immune cells and osteoporosis

### 3.1 From osteoimmunology to immunoporosis: immune imbalance caused bone loss regulate bone remodeling plays an important role in regulating bone health and homeostasis

The relationship between the immune system and the bone system has long been studied. In 2000, Arron et al. proposed the concept of “osteoimmunology” and pointed out that there is an intricate interaction between the immune system and the bone system (42). Since then, immune-related factors have gradually become a hot research topic in metabolic bone diseases. In fact, the regulatory between the immune system and bone is bidirectional (43). On the one hand, as immune cells form in the bone marrow, the bone system exerts an important influence on the generation, function and regulation of the immune system through affecting the generation and differentiation of hematopoietic stem cells (HSCs) and the secretion of cytokines (44). On the other hand, the immune system regulates bone health and homeostasis by modulating the differentiation of OBs and OCs, as well as the inflammatory responses (45). It has been shown previously that the RANKL/RANK/OPG pathway was thought to act as a link between immune system and bone (46). Activated T cells release RANKL, a crucial cytokine in osteoclastogenesis and bone resorption, which binds to RANK and then regulates bone remodeling by activating a series of signal transduction pathways.

Recently, Srivastava et al. have coined the term “immunoporosis” to highlight the role of immune cells in the pathology of OP (47). Immune OP provides a good perspective for understanding the complex interactions between the immune system and the skeletal system. It enables us to use immune knowledge to explain the pathological mechanism of OP and provide important clues for the development of new treatments and drugs for OP in the future. A summary of the mechanisms by which immune cells regulate bone metabolism, as discussed in this article, is provided in **Table 1**.

**Table 1:** Summary of the mechanism by which immune cells regulate bone metabolism.

Immune cells	Subtype	Regulatory mechanism	Effects on bone metabolism
T lymphocytes	Th17	Pro-inflammatory cytokine secretion via STAT3 (e.g., TNF-α, IL-17, IL-6); RANKL activation; OPG inhibition.	OCs production↑ Bone resorption↑
	Tregs	Anti-inflammatory cytokine secretion via Foxp3 (e.g., IL-4, IL-10, and TGF-β); RANKL inhibition.	OBs production↑; Bone formation↑
B lymphocytes	B cells	OPG production↑; OBs differentiation inhibition via ERK and NF-κB; OCs differentiation via G-CSF secretion (Inflammatory condition)	Bone resorption↓(Physiological condition) Bone resorption↑(Inflammatory condition)
	Regulatory B lymphocytes (Bregs)	Anti-inflammatory cytokine secretion (e.g., IL-35, IL-10, and TGF-β1)	Bone resorption↓
Macrophages	M1 polarization	Pro-inflammatory cytokine secretion (e.g., IL-1, IL-6, IL-12, TNF-α, NO, ROS)	OCs production↑; Bone resorption↑
	M2 polarization	Anti-inflammatory cytokine secretion (e.g., IL-10, TGF-β); pro-osteogenic molecules secretion (e.g., BMP-2)	OBs production↑; Bone formation↑
Dendritic cells (DCs)	Conventional DCs 1 (cDC2)	High expressing CD80/CD86 for antigen presentation; promote osteoclastogenesis indirectly through T cell activation	OCs production↑; Bone resorption↑
	Conventional DCs 2 (cDC2)	OPG secretion; TGF-β secretion	Bone resorption↓
Neutrophils	--	Phagocytosis, degranulation, and neutrophil extracellular trap (NET) formation	Bi-directional regulation
Mast cells	--	Estrogen deficiency; pro-osteogenic effects via PDGF secretion	Bi-directional regulation
Natural Killer cells	--	Pro-osteogenic molecules secretion (e.g., RANKL, M-CSF); IL-15-activated NK cells eliminate OCs via LFA-1/ICAM-1 and DNAM-1/CD155	Bi-directional regulation
Natural Killer T cells (NKT)	invariant NKT (iNKT)	T cells/macrophages activation via IFN-γsecretion; enhanced TNF-α secretion; directly control OCs differentiation	OCs production↑; Bone resorption↑
	CD56bright NKT	Enhanced trafficking to inflammatory sites	

Mechanistic insights and functional annotations are based on findings discussed in Section 3 of the main text. Detailed citations can be found in Section 3.

STAT3, signal transducer and activator of transcription 3; RANKL, receptor Activator of Nuclear Factor-κB Ligand; OPG, osteoclastogenesis inhibitory factor; OCs, Osteoclasts; OBs, Osteoblasts; ERK, extracellular regulated protein kinases; NF-κB, nuclear factor kappa-B; G-CSF, granulocyte colony-stimulating factor; TGF-β, transforming growth factor-β; BMP-2, bone morphogenetic protein 2; PDGF, platelet-derived growth factor; M-CSF, macrophage-colony stimulating factor.

### 3.2 T lymphocytes and osteoporosis

In particular, T lymphocytes (T cells) play pivotal roles in the regulation of bone health.

T cells are the core cells of immune regulation, and naïve T cells can differentiate into Treg cells, Th17 cells and other subsets after stimulation by exogenous antigens, and can produce different immune effects by secreting different characteristic cytokines (48). Th17 cells are essential core links in estrogen deficiency-induced bone loss (49). In addition, Treg cell deficiency and inactivation are associated with some chronic inflammatory diseases, and Treg cells can regulate OCs formation and prevent bone resorption by secreting IL-4, IL-10, and TGF-β (50).

The imbalance between Th17 cells and Treg cells constitutes a pivotal mechanism underlying OP pathogenesis (50). Th17 cells secrete IL-17 to activate RANKL expression in bone marrow stromal cells while suppressing osteoprotegerin (OPG) production, thereby disrupting the RANKL/OPG equilibrium (51). This ratio imbalance amplifies OCs differentiation through NF-κB signaling cascades (52). Experimental studies confirm that phosphorylation of STAT3, the Th17-specific transcription factor, upregulates Cathepsin K expression to enhance osteoclastic bone resorption activity (53).

Treg cells employ Foxp3-dependent pathways to secrete TGF-β and IL-10, directly inhibiting RANKL-induced osteoclastogenesis (54). Their surface molecule CTLA-4 interacts with CD80/CD86 on dendritic cells, blocking costimulatory signaling and suppressing pro-osteoclastogenic factors like TNF-α (55). Under physiological conditions, Treg cells maintain bone equilibrium by balancing Foxp3/RORγt transcriptional factor expression in CD4+ T cells, thereby counteracting Th17-mediated bone destruction (54). Postmenopausal estrogen deficiency reduces bone marrow Treg population and functional competence, concurrently promoting Th17 differentiation into IL-23 receptor-high subsets (56). This immunologic shift activates JAK2/STAT3 pathway to induce OBs apoptosis and enhances OCs precursor sensitivity to M-CSF, ultimately driving bone mass loss (57). Elucidation of this regulatory network provides theoretical foundation for immunologically targeted interventions in OP management.

### 3.3 B lymphocytes and osteoporosis

B lymphocytes, pivotal components of humoral immunity, develop from HSCs precursors in bone marrow and primarily function through antibody secretion to neutralize pathogens while enhancing effector functions of other immune cells (58). Emerging research increasingly implicates B lymphocytes in bone remodeling pathologies. Critically, these cells produce approximately 50% of bone marrow-derived OPG, which competitively inhibits RANKL binding to suppress osteoclastogenesis and prevent excessive bone resorption (59). Conversely, B lymphocytes secrete C-C motif chemokine ligand 3 (CCL3) and tumor necrosis factor (TNF) that impair OBs differentiation via extracellular signal-regulated kinase (ERK) and nuclear factor kappa-light-chain-enhancer of activated B cells (NF-κB) pathways (60).

The osteoimmunological impact of B lymphocytes is context-dependent. Inflammatory conditions polarize B cells toward pro-osteolytic phenotypes: Activated B lymphocytes secrete granulocyte colony-stimulating factor (G-CSF) that expands OCs progenitor pools, while concurrent RANKL and G-CSF production drives OCs proliferation and differentiation, ultimately driving progressive osteopenia (61, 62). Clinically, postmenopausal osteoporotic women exhibit significantly reduced CD19+ B lymphocyte frequencies versus healthy controls, underscoring the pivotal contributions of B lymphocytes to OP pathogenesis through immunomodulatory and osteometabolic pathways (63).

Beyond conventional antibody-secreting subsets, regulatory B lymphocytes (Bregs) counterbalance OCs activity through anti-inflammatory mediators (64). IL-35 promotes Breg differentiation via STAT1/STAT3 signaling (65); IL-10 directly impedes osteoclastogenesis by disrupting Ca²^+^ mobilization and NFATc1 signaling in precursors while enhancing OBs differentiation through miR-7015-5p downregulation (66, 67); and TGF-β1 stimulates osteogenesis via SMAD/MAPK-mediated Runt-related transcription factor 2 (RUNX2) upregulation while suppressing osteolytic genes encoding tartrate-resistant acid phosphatase (TRAP) and cathepsin K (68). Thus, in TGF-β1-deficient osteoporotic microenvironments, sustained RANKL expression on OBs coupled with attenuated RUNX2-directed osteogenesis collectively drives pathologic bone remodeling toward resorption.

### 3.4 Macrophages and osteoporosis

As pivotal innate immune cells, macrophages orchestrate bone immunometabolic homeostasis through their remarkable phenotypic plasticity, dynamically transitioning between polarization states to regulate tissue balance (69). Proinflammatory cytokines including IL-1β, IL-6, IFN-γ, and TNF-α, along with bacterial components like LPS, drive M1 polarization of macrophages (70). These classically activated cells release inflammatory mediators such as IL-1β, IL-6, IL-12, TNF-α, nitric oxide (NO), and reactive oxygen species (ROS), which directly or indirectly stimulate osteoclastogenesis and enhance bone resorption (71). When sustained, this M1-dominant state propagates chronic inflammation, triggering a compensatory shift toward M2 polarization via anti-inflammatory cytokines (e.g., IL-4, IL-10, IL-13) to restore tissue equilibrium (72).

M2-polarized macrophages contribute to tissue repair through efferocytosis of apoptotic cells, secretion of anti-inflammatory cytokines like IL-10, and production of pro-osteogenic molecules such as bone morphogenetic protein 2 (BMP-2) and transforming growth factor β (TGF-β) (73). These factors induce bone marrow mesenchymal stem cells (BMSCs) differentiation into mature OBs, accelerating bone regeneration (74). Notably, elevated M1/M2 macrophage ratios in OP models underscore macrophage repolarization as a promising therapeutic target (75).

Recent mechanistic studies reveal novel regulatory pathways: Huang et al. demonstrated that M2-derived extracellular vesicles (M2-EVs) reprogram osteoclast precursors (OCPs) into M2-like macrophages, rebalancing the OC-macrophage axis to attenuate pathological bone loss (76). This strategy offers a bone-targeting approach for OP therapy. Complementary work by Qin et al. showed that bilobalide—a bioactive constituent of Ginkgo biloba—promotes dose-dependent M2 polarization while suppressing RANKL-induced osteoclastogenesis through SIRT3 upregulation and NF-κB pathway inhibition, revealing another promising intervention for OC-mediated bone loss (77).

Collectively, these advances position M2 macrophages as central orchestrators of osteogenic activity, with macrophage phenotype modulation representing a compelling therapeutic frontier for OP management.

### 3.5 Others immune cells and osteoporosis

#### 3.5.1 Dendritic cells

Dendritic cells (DCs), specialized antigen-presenting cells (APCs), serve as pivotal initiators of adaptive immune responses. Distributed ubiquitously, they continuously monitor danger signals via pattern recognition receptors (PRRs) while constitutively expressing high levels of major histocompatibility complex class II (MHC-II) molecules and co-stimulatory receptors (CD80/CD86), essential for antigen presentation. Emerging research reveals DCs as critical regulators of bone homeostasis and pathological remodeling, demonstrating striking functional duality (78).

Under RANKL and M-CSF stimulation, DCs exhibit significantly greater efficiency in transdifferentiating into OCs than monocytes, with transcriptomic analyses confirming fewer regulatory steps required for this conversion, suggesting close developmental proximity between these lineages (79). Activated DCs promote osteoclastogenesis indirectly through T cell activation—particularly Th17 polarization—triggering IL-17 and TNF-α secretion that induces RANKL production in bone stromal cells (78, 80). Furthermore, DCs and T cells form pathological aggregates that exacerbate osteolytic conditions including synovitis and periodontitis (81, 82). Significantly, a self-amplifying loop emerges wherein newly formed OCs chemoattract additional DCs to bone resorption sites through chemokine signaling (83).

Conversely, DCs counterbalance bone destruction via cDC2-derived OPG—a decoy receptor that competitively inhibits RANKL binding to suppress OCs activation (59). Under specific conditions, DCs also secrete TGF-β and other anti-resorptive molecules that mitigate inflammatory bone loss (84). Crucially, DCs function as dynamic immunoregulatory buffers that fine-tune the osteolytic-osteogenic equilibrium during OP initiation (85).

#### 3.5.2 Neutrophils

Neutrophils—polymorphonuclear granulocytes constituting 40%-60% of circulating leukocytes—serve as primary sentinels of innate immunity. Their production is regulated by G-CSF and granulocyte-macrophage colony-stimulating factor (GM-CSF), with mature cells rapidly recruited to inflammatory sites where they orchestrate local microenvironments through phagocytosis, degranulation, and neutrophil extracellular trap (NET) formation (86). Beyond these canonical functions, neutrophils synthesize C-X-C and C-C chemokines facilitating crosstalk with bone cells and other cellular constituents (87).

Emerging research reveals neutrophils modulate skeletal metabolism through multiple pathways, where their senescence or dysfunction directly/indirectly impacts OCs, OBs, and MSCs (88, 89). Neutrophils exacerbate bone resorption by secreting RANKL, generating ROS, and releasing pro-inflammatory cytokines that potentiate osteoclastogenesis (90, 91). Simultaneously, they recruit pro-osteoporotic cells like Th17 lymphocytes via chemokines (IL-8, IL-17), further amplifying osteoclastic activity (92). Conversely, neutrophils enhance bone formation by inducing OBs expression of mineralization markers including ALP and osteocalcin, thereby promoting mineral deposition (93). They also regulate MSC osteogenic differentiation; though notably, *in vitro* studies demonstrate neutrophils inhibit MSC-mediated extracellular matrix production while G-CSF-induced neutrophil expansion triggers ROS-mediated apoptosis in MSCs and OBs, collectively attenuating osteogenesis (94, 95).

#### 3.5.3 Mast cells

Mast cells (MCs) exhibit unique spatial positioning in osteoporotic bone, predominantly accumulating within endosteal marrow regions while closely adjacent to OCs surfaces (96). The resulting osteometabolic imbalance manifests initially as enhanced endosteal resorption within metaphyseal compartments, subsequently progressing to affect cortical endosteal surfaces in epiphyseal and diaphyseal regions. Estrogen deficiency constitutes a key permissive condition for MC-mediated osteopathology, as evidenced by significant MC expansion in OVX rat marrow compared to sham controls (97). Mechanistically, MC-deficient mice resist OVX-induced bone loss through suppressed OCs numbers and activity (98). Critically, mast cell accumulation in OVX mice directly correlates with elevated OCs numbers (99), exhibiting frequent spatiotemporal co-localization with OCs (96). Consequently, estrogen deficiency functions as a crucial molecular switch unlocking mast cell-mediated osteolytic activity.

Compared to OC interactions, MC influences on OBs remain less characterized. Lind et al. demonstrated elevated bone mass, formation markers, and mineralization rates in female mice lacking the MC protease chymase Mcpt4, suggesting chymase inhibits osteogenesis (100). Further supporting an anti-osteogenic role, MCs suppress OB differentiation via IL-1 secretion (96). However, conflicting reports indicate pro-osteogenic effects: Turner et al. identified MC-derived platelet-derived growth factor (PDGF) promoting stromal cell differentiation toward osteoprogenitors (101), and MC-deficient mice exhibit reduced OBs activity (102), revealing context-dependent regulatory duality.

#### 3.5.4 Natural Killer cells

Natural killer (NK) cells, traditionally derived from bone marrow HSCs, now demonstrate maturation capacity within secondary lymphoid tissues (SLTs) where they acquire adaptive features including memory functions (103). Beyond canonical tumor-lytic activity, NK cells eliminate virus-infected and stress-altered cells (104, 105) through balanced signaling via activating/inhibitory receptors (106). These lymphocytes further sculpt local microenvironments by mediating antibody-dependent cellular cytotoxicity (ADCC) and secreting pro-inflammatory mediators (e.g., IFN-γ, chemokines) that regulate neighboring immune cells (107).

Notably, NK cells exhibit context-dependent functional duality in inflammatory osteopathies. In RA, synovial-infiltrating NK cells highly express osteoclastogenic factors RANKL and M-CSF—levels further augmented by IL-15 (108). Clinically, RA synovial fluid NK cells drive monocyte differentiation into functional OCs, with IL-15 conferring enhanced resorptive capacity (109). Correspondingly, NK cell depletion in collagen-induced arthritis (CIA) models significantly attenuates bone destruction (109). Conversely, IL-15-activated NK cells eliminate OCs via LFA-1/ICAM-1 and DNAM-1/CD155 contact-dependent cytolysis, an effect abrogated by receptor blockade (110). This phenotypic plasticity indicates NK cell OC-regulatory behavior depends critically on microenvironmental cues. Supporting clinical significance, bioinformatic analyses reveal expanded T and NK cell frequencies in PMOP bone (111).

#### 3.5.5 Natural Killer T cells

Natural killer T (NKT) cells constitute critical regulators of skeletal metabolism, exhibiting a hybrid T-NK phenotype that enables both cytotoxic clearance of compromised cells (112) and cytokine-mediated modulation of T/B cell immunity (113, 114) and myeloid functions (115). Recent advances reveal invariant NKT (iNKT) subsets directly control OCs differentiation through specialized pathways (116). Within rheumatoid RA pathology, synovial NKT cells expand dramatically—comprising ≤ 20% of lymphocytes (117)—with CD56bright subsets exhibiting enhanced chemokine receptor-mediated trafficking to inflammatory sites (118). These infiltrating NKT cells activate monocytes to promote osteoclastogenesis (117), while concurrently secreting RANKL and M-CSF (potentiated by IL-15) (119). Significantly, NKT-derived IFN-γ stimulates T cells/macrophages to amplify TNF-α release (120), which subsequently drives osteoprogenitor differentiation through RANKL-dependent mechanisms while inducing OBs to secrete additional RANKL/M-CSF (121, 122). This multi-layered cooperativity establishes NKT cells as principal orchestrators of inflammatory bone loss.

Taken together, these findings indicate that both innate and adaptive immune cells persistently shape OCs and OBs functions. This interaction is crucial for deciphering physiological bone metabolic equilibrium and pathological remodeling processes in OP.

## 4 Gut microbiota influences immune system

The GM orchestrates maturation and functional development of the early human immune system from birth. Gnotobiotic models provide definitive evidence: studies comparing GF and specific pathogen-free mice reveal significant developmental impairments in gut-associated lymphoid tissue and defective formation of isolated lymphoid follicles—critical sites for IgA response induction (123). Critically, the GM further modulates systemic immunity and self-antigen responsiveness, with microbiota alterations reported across preclinical and clinical models of chronic diseases underscoring its influence on immune dysregulation during pathogenesis (124).

GM-immune crosstalk in extraintestinal organs operates through three interconnected mechanisms (125): firstly through direct cellular interactions where microbes engage immune cells via surface adherence or phagocytic uptake, and bind pattern-recognition receptors on mucosal epithelia and macrophages to trigger pro-inflammatory cascades that amplify immune activity; mechanistically through metabolite-mediated signaling where SCFAs and other immunomodulatory molecules directly govern immune cell proliferation, differentiation, and effector function; furthermore through barrier-dependent segregation wherein epithelial structures physically and chemically sequester luminal communities to maintain host-commensal homeostasis by preventing aberrant lymphocyte activation.

Thus, the immune-GM axis pivots on these molecular pathways, warranting detailed examination of their collective impact on immune regulation.

### 4.1 Gut microbiota and Immune cells

The GM primarily colonizes the gastrointestinal tract, with highest density in the colorectum, and is predominantly composed of Firmicutes and Bacteroidetes (collectively representing ~90% of total abundance), alongside minor constituents including Proteobacteria and Actinobacteria (126). Gut homeostasis is characterized by dominance of obligate anaerobes from Firmicutes and Bifidobacteriaceae, whereas expansions in facultative anaerobes of the Enterobacteriaceae family commonly signify dysbiosis (127). Functionally, Firmicutes abundance positively correlates with calcium absorption (128), while Bacteroidetes orchestrate immune equilibrium by restoring Th1/Th2 balance, redirecting lymphoid organogenesis in gnotobiotic animals, and directing cellular/physical maturation of the developing immune system (129).

The intestinal epithelium establishes sophisticated physical and chemical barriers that spatially segregate GM from immune cells, preventing excessive immune activation while concurrently functioning as a critical communication interface between these compartments (130). A subset of bacterial species adhere tightly to small intestinal epithelial cells (IECs), exploiting this unique niche colonization to induce specific gene expression programs in IECs that subsequently promote immune cell differentiation. This is exemplified by *segmented filamentous bacteria* (SFB), whose attachment to IECs triggers epithelial production of serum amyloid A (SAA), thereby driving Th17 response differentiation and enhancing antimicrobial defense against bacterial pathogens (131).

While most GM remain non-adherent to the intestinal epithelium, immune cells and IECs deploy PRRs for microbial surveillance, recognizing bacterial components through Toll-like receptors (TLRs) and NOD-like receptors (NLRs) (132, 133). Specific bacterial components—including lipopolysaccharide (LPS), flagellin, and *Bacteroides fragilis* capsular polysaccharide A (PSA)—are detected by these PRRs, orchestrating downstream innate immune signaling cascades.

#### 4.1.1 Lipopolysaccharide

Gram-negative bacterial LPS exemplifies this signaling cascade: initially complexing with LPS-binding protein (LBP), it transfers to CD14 on myeloid cells before engaging the TLR4-MD2 complex. This binding induces TLR4 dimerization and conformational change, triggering dual signaling axes through myeloid differentiation factor 88 (MyD88) and IL-1R-associated kinase (IRAK) (134). Downstream activation of NF-κB and MAPK pathways drives production of pro-inflammatory mediators (IL-1β, IL-6, TNF-α, NO), promotes M1 macrophage polarization (70), and enhances DCs antigen presentation via upregulated MHC-II and co-stimulatory molecules (CD80/86) (135), while concurrently stimulating neutrophil recruitment through CXCL1/2 chemokine release (136). Concomitantly, the TLR4/TRIF axis phosphorylates TANK-binding kinase 1, which phosphorylates interferon regulatory factor 3 to drive type I interferon (IFN-β) production (137). This IFN-I signaling potentiates cytotoxic programs in NK cells and CD8^+^ T lymphocytes (138, 139).

#### 4.1.2 Flagellin

Flagellin from Gram-negative bacteria (including invasive pathogens) is primarily recognized by lamina propria-resident DCs with high TLR5 expression, triggering MyD88-dependent signaling to generate abundant pro-inflammatory cytokines (140). Select pathogens like *Salmonella* further leverage type III secretion systems to translocate flagellin into host cytosol (141). During intracellular delivery, flagellin is initially sensed by NAIP5/6 proteins (NLR family apoptosis inhibitory proteins), which recruit NOD-like receptor NLRC4 to assemble inflammasomes. This cascade activates caspase-1, cleaving pro-interleukin-1β/18 into mature IL-1β/IL-18 to induce pyroptosis (142).

Intriguingly, commensal-derived flagellins exhibit distinct immunomodulatory properties, as demonstrated by Clasen et al. (143). While binding TLR5 with comparable affinity to pathogenic flagellins, these “silent flagellins” evade inflammatory activation—revealing novel recognition paradigms within innate immunity.

Beyond innate sensing, TLR5 coordination extends to humoral immunity: Intestinal epithelial TLR5 engagement activates NF-κB, stimulating B-cell activating factor and proliferation-inducing ligand secretion (144). These cytokines drive B-cell differentiation into IgA-producing plasma cells that neutralize pathogens (145).

#### 4.1.3 Fragilis polysaccharide A

Bacteroides fragilis, a commensal bacterium inhabiting the colonic mucosa, PSA—an immune-tolerant symbiosis factor conferring therapeutic benefits against intestinal inflammation and systemic immune-mediated disorders like experimental autoimmune encephalomyelitis (EAE) (146, 147). PSA engages TLR2/TLR1 heterodimers on DCs to induce IL-10^+^/TGF-β^+^ Tregs while suppressing pro-inflammatory cytokines, establishing an immunosuppressive niche (129, 148).

Ertürk-Hasdemir et al. demonstrated that PSA-mediated EAE protection requires functional TLR2/TLR1 signaling (149). Concurrently, PSA activates Dectin-1 lectin receptors, triggering Syk kinase phosphorylation that converges with TLR2 signaling at PI3K (149). Notably, ablation of either receptor ablates PI3K-dependent Akt phosphorylation, disrupting anti-inflammatory gene programs.

Mechanistically, PSA-driven Akt phosphorylation inhibits GSK3β, preventing NF-κB/CBP complex formation while promoting CREB-dependent transcription of immunosuppressive mediators (IL-10, MHC-II, ICOS-L) (150). This epigenetic reprogramming initiates Foxp3^+^ IL-10^+^ Treg expansion, completing a multi-receptor immunosuppressive circuit (151).

### 4.2 Gut microbiota metabolites and Immune cells

Beyond the GM themselves, their metabolites significantly contribute to intestinal homeostasis and inflammation regulation by modulating immune responses.

#### 4.2.1 Short-chain fatty acids

SCFAs—predominantly acetate, propionate, and butyrate—are abundantly produced in the colon through microbial fermentation of dietary fiber (152). SCFAs suppress inflammation and carcinogenesis by directly or indirectly inhibiting histone deacetylase (HDAC) activity, primarily via activation of G protein-coupled receptors (GPCRs), thereby influencing immune cell differentiation and function (152).

Specifically, butyrate and propionate enhance histone H3 acetylation, opening the promoters of the Foxp3 and IL-10 genes to drive Treg differentiation (153, 154). Concurrently, they activate the aryl hydrocarbon receptor (AhR), synergistically augmenting the anti-inflammatory effects of tryptophan metabolites (155). Functional evidence demonstrates that propionate mediates its protective effects through GPR43 signaling: GPR43-knockout mice with chronic inflammation exhibit reduced intestinal Tregs, elevated Th17 cell proportions, and aggravated experimentally induced colitis compared to wild-type mice following propionate administration (156). This establishes that propionate inhibits HDAC via GPR43 to enforce anti-inflammatory activity against T cell-driven colitis.

In innate immunity, butyrate-activated GPR109A signaling induces arginase-1 (Arg1) and IL-10 expression in macrophages and DCs, polarizing them toward an anti-inflammatory phenotype that promotes IL-10-producing CD4^+^ T cells and Treg differentiation in the colon (157). Furthermore, acetate binding to GPR43 on IECs stimulates potassium efflux and membrane hyperpolarization, triggering NLRP3 inflammasome activation. The consequent release of IL-18 contributes to intestinal homeostasis and colitis prevention (158).

#### 4.2.2 Tryptophan

Tryptophan, an essential dietary amino acid, serves as a precursor for diverse metabolic transformations through direct microbial metabolism or conversion via the kynurenine pathway into indole derivatives, kynurenine (Kyn), and downstream metabolites (159). The AhR—a ligand-activated transcription factor expressed across immune cell populations—represents a central mechanism whereby tryptophan metabolites regulate immunity (160). Indole-3-propionic acid (IPA) activates AhR signaling in CD4^+^ T cells, driving differentiation of RORγt^+^ Tregs (161). Concurrently, AhR inhibits Th17 differentiation by suppressing STAT3 phosphorylation, consequently reducing IL-17A secretion (162). Substantiating AhR’s critical role in gut immunity, intestinal Tregs exhibit significantly higher AhR expression than Tregs at other anatomical sites (163).

Regarding innate immunity, *in vitro* studies demonstrate that tryptophan metabolites inhibit inflammatory responses by suppressing histamine production in macrophages (164). Kyn further induces M2 macrophage polarization via AhR activation, promoting expression of anti-inflammatory mediators IL-10 and arginase-1 (Arg1) (165). This mechanism confers protection against septic colonic injury through the PPARγ/NF-κB axis (166). The downstream Kyn metabolite 3-HAA suppresses dendritic cell maturation by downregulating CD40/CD80/CD86/I-A expression and reducing IL-6, IL-12, and TNF-α production in LPS-stimulated bone marrow-derived DCs (BMDCs). It concurrently inhibits phospho-JNK and phospho-p38 levels in both DC2.4 cells and BMDCs, collectively demonstrating that 3-HAA impairs CD4^+^ T cell activation and proliferation through DC suppression (167).

#### 4.2.3 Secondary bile acids

Secondary bile acids (SBAs), including deoxycholic acid (DCA), lithocholic acid (LCA), and ursodeoxycholic acid (UDCA), are primarily generated by GM (e.g., *Clostridium* spp.) via 7α-dehydroxylation of primary bile acids (168). These metabolites regulate immune cell function primarily through activation of the farnesoid X receptor (FXR) and G protein-coupled bile acid receptor 1 (TGR5) (169).

SBAs interact with TGR5 in macrophages, inhibiting NLRP3 inflammasome assembly and suppressing NF-κB signaling through the cAMP-PKA pathway (170). This promotes IL-10 secretion and induces an M2-polarized phenotype (171, 172). The SBA 3β-hydroxydeoxycholic acid (isoDCA), produced via microbial epimerization of cholic acid, attenuates immunostimulatory properties in DCs through FXR signaling (173). This enhances Foxp3 induction, thereby promoting Treg generation and differentiation. Supporting this mechanistic insight, engineered minimal microbial consortia containing *Bacteroides* strains expanded intestinal RORγt^+^ Treg populations (173).

Notably, isoallolithocholic acid (isoalloLCA) amplifies Treg differentiation through mitochondrial ROS generation, elevating Foxp3 expression (174). Beyond Treg modulation, 3-oxolithocholic acid (3-oxoLCA) directly binds the transcription factor RORγt to inhibit Th17 cell differentiation (174). Thus, bile acid metabolites control host immunity by directly balancing Th17 and Treg responses. Crucially, RORγ^+^ Treg homeostasis depends on the collective intestinal bile acid pool rather than individual SBAs, underscoring the host-microbial biliary network’s role in gut immune homeostasis (175).

In the liver, SBAs reduce CXCL16 expression on sinusoidal endothelial cells, limiting hepatic recruitment of CXCR6-expressing NKT cells (176). Consistent with this immunomodulatory function, selective FXR agonists significantly suppress liver tumor growth in murine models (177, 178). Notably, an obeticholic acid (OCA, a clinically approved FXR agonist) nanoemulsion prepared via ultrasonication demonstrated superior efficacy to oral free OCA (178).

### 4.3 Gut microbiota and Intestinal epithelial cells

As the intermediary layer of the gut barrier, IECs physically separate the underlying lamina propria from luminal pathogenic threats and commensal microorganisms. This compartmentalization is essential for executing protective immune functions. Luminal antigens and microbiota-derived signals transmit information to IECs, mediating adaptation to intestinal environmental shifts through mucosal barrier modulation. Gut microbial signals engaging IECs are broadly categorized into three classes: bacteria themselves, bacterial components, and bacteria metabolites (125).

#### 4.3.1 Bacteria themselves and bacterial components

As previously described, IECs express TLRs and NLRs for immune surveillance (179). These receptors rapidly detect bacterial components like LPS and flagellin, driving epithelial proliferation while inducing expression and secretion of cytokines, antimicrobial molecules, and mucus in IECs (125). The Regenerating Islet-derived 3 (Reg3) protein family—secreted by Paneth cells—constitutes a critical class of antimicrobial effectors that spatially segregate GM from the epithelial surface (180). In the small intestine, Paneth cells secrete antimicrobial molecules (including Reg3 proteins and α-defensins) via TLR/MyD88 signaling, thereby reducing pathogen colonization and maintaining microbial ecology (181). Within the colon, LPS and flagellin induce goblet cells to secrete mucin-2 (MUC2) through TLR ligand engagement (182). Critically, Myd88 deficiency in IECs reduces MUC2 expression and Reg3γ production, exacerbating susceptibility to colitis and impairing resistance to *Salmonella Typhi* or *Citrobacter rodentium* infections (180, 183).

Cytosolic NLR receptors further uphold mucosal barrier integrity. *Lactobacillus reuteri* (*L. reuteri*) activates NOD2 signaling in IECs to stimulate Paneth cell secretion of antimicrobial peptides (e.g., α-defensins), indirectly suppressing Th1/Th17 responses while enhancing Treg function (184). Moreover, NLRP6 promotes mucin exocytosis by goblet cells through autophagy-mediated vesicle trafficking, which crucially restricts colonization by pathobionts like *Prevotellaceae* and candidate phylum TM7 (185, 186).

#### 4.3.2 Bacteria metabolites

SCFAs, particularly butyrate, serve not only as the preferred energy substrates for IECs but also as critical modulators of IEC and immune cell physiology. They play pivotal roles in maintaining epithelial integrity and repairing mucosal damage (187). Through activation of GPR41, GPR43, and GPR109A expressed on IECs, SCFAs induce expression of tight junction proteins (e.g., ZO-1, occludin, claudin-1), thereby reducing intestinal permeability (188). Notably, butyrate primarily signals via GPR109A on IECs or lamina propria dendritic cells, whereas acetate and propionate function through GPR43/GPR41 activation (189). Additionally, SCFAs enhance mucosal barrier function by promoting histone acetylation at the MUC2 locus to increase mucus layer thickness while simultaneously stimulating Paneth cell exocytosis of AMPs (190). This coordinated action—fortifying physical barrier integrity through enhanced intercellular junctions and reinforcing chemical defenses via AMP secretion—demonstrates how SCFAs regulate IEC-microbial crosstalk to prevent pathogen invasion.

AHR is constitutively expressed across IECs, enabling detection of indole and its derivatives derived from tryptophan metabolism. Animal studies demonstrate that AHR deficiency in IECs significantly reduces MUC2 and carbonic anhydrase 4 (Car4) expression, consequently compromising resistance to pathogenic infections (191). Additionally, AHR modulates the Wnt/β-catenin signaling pathway in IECs, thereby regulating the specification of epithelial and crypt stem cells (191). Crucially, AHR governs group 3 innate lymphoid cell (ILC3) development, where indole metabolites stimulate ILC3s to secrete IL-22—a cytokine essential for enhancing barrier function through induction of AMPs production and intestinal infection defense (192). Intriguingly, indole compounds concurrently activate the pregnane X receptor (PXR), upregulating junctional proteins including occludin and claudin-1 through ligand binding (193). These findings establish that indole-mediated barrier enhancement operates through coordinated AHR and PXR activation in IECs, playing indispensable roles in maintaining epithelial barrier integrity.

Bile acids exert dual effects on intestinal barrier integrity (194). Primary bile acids (e.g., cholic acid and chenodeoxycholic acid) exert cytotoxic effects on IECs (195), whereas secondary bile acids (e.g., DCA and LCA) enhance barrier function by modulating expression of tight junction proteins including claudin-1, claudin-4, and occludin (196). Specifically, DCA inhibits IEC proliferation and wound healing through FXR activation (197). Additionally, DCA stimulates TGR5 on enteroendocrine cells, triggering release of 5-hydroxytryptamine (5-HT) and calcitonin gene-related peptide (CGRP) to promote colonic motility (198).

Collectively, GM and their bioactive metabolites continuously shape IEC activity and proliferation. This dynamic regulation is indispensable for maintaining barrier competence—preventing bacterial translocation and mucosal damage while ensuring efficient pathogen exclusion.

## 5 Immune changes resulting from disruption of the microbiome composition can affect bones

The human GM encompasses approximately 1,000 species across 28 distinct phyla—exceeding human cell counts while expressing ~100-fold more genes than the human genome—illustrating remarkable complexity and diversity (199). Consequently, compositional shifts in this microbial community can exert either beneficial or detrimental effects on human health (200).

Animal studies reveal that GF mice—exhibiting GM dysbiosis—display reduced CD4^+^ T cells and OCs alongside diminished TNF-α and IL-1 expression compared to conventional counterparts. Remarkably, GF mice colonized with normal microbiota restored bone mass to physiological levels (12). Similarly, colonization with microbiota-derived SCFAs normalized bone mass in GF mice, potentially mediated via GM-induced insulin-like growth factor 1 (IGF-1) production that stimulates osteogenesis (201). Clinically, malnourished and growth-stunted children exhibit compromised microbiota relative to healthy controls (202). Critically, administering two bacterial strains (*Ruminococcus* and *Clostridium symbiosum*) rectified growth impairment in mice transplanted with malnourished children’s microbiota (202). A randomized double-blind trial demonstrated that six-month multi-species probiotic supplementation significantly decreased bone-specific ALP and CTX levels—key biomarkers of bone turnover—in PMOP patients, attenuating bone resorption (203). Collectively, these findings demonstrate that microbiota dysbiosis triggers immune alterations impacting skeletal integrity and development, whereas probiotic interventions can restore microbial ecology to promote bone health—though precise mechanistic details remain elusive.

Beyond microbial complexity, limited culturability poses fundamental challenges to delineating GM regulatory mechanisms. Of approximately 1000 resident microbial species, fewer than 40% are currently culturable *in vitro*, leaving >60% of functional potentials unexplored. The discovery of “silent flagellins” —mutated bacterial proteins evading innate immune recognition—reveals novel microbial evasion strategies while underscoring critical knowledge gaps in host-microbe interactions (143). Advancements in molecular tools and technologies—such as metagenomics, metabolomics, lipidomics, and metatranscriptomics—have enabled the gradual deciphering of complex interactions between GM, their metabolites, and bone metabolism. These developments allow us to extend beyond classical immunology, thereby deepening our understanding of the gut-bone axis. All microbial species discussed herein and their mechanisms of bone metabolism regulation are summarized in **Table 2**.

**Table 2:** Summary of the major functions of gut microbiota in the gut-bone axis.

Gut microbiota species	Phylum	Major functions in the gut-bone axis	References
Prevotellaceae	Bacteroidetes	Positive correlations with the development of systemic inflammation and the onset of RA	(13)
Fusobacterium nucleatum	Fusobacteria	M1 macrophage polarization modulation via the AKT2 signaling pathway, exacerbating colonic inflammation	(15)
Akkermansia muciniphila	Verrucomicrobiota	Bone formation promotion and bone absorption inhibition by releasing EVs into bone tissue	(17)
Lactobacillus acidophilus	Firmicutes	Relative abundance increasing (e.g., Lactobacillus, Allobaculum, and UCG-005); Metabolites production (e.g., indicaxanthin, chlorogenic acid, and 3-hydroxymelatonin) ; scalcium absorption improvement; regulation of Th17/Treg immune homeostasis	(20, 216)
Lactobacillus rhamnosus GG	Firmicutes	Regulation of Th17/Treg immune homeostasis	(25)
Butyricicoccus pullicaecorum	Firmicutes	Regulation of Th17/Treg immune homeostasis	(26)
Filamentous bacteria	Bacillota	Th17 differentiation promotion via SAA production	(131)
Salmonella	Enterobacteriaceae	Flagellin translocation via type III secretion systems	(141)
Bacteroides fragilis	Bacteroidetes	Induction of anti-inflammatory factor production (e.g., TGF-β, IL-10) in DCs and Tregs.	(129, 148)
Lactobacillus reuteri (L. reuteri)	Firmicutes	Antimicrobial peptides secretion via NOD2 signaling; hormones production (e.g., serotonin, GIP, PYY, vasopressin and luteinizing hormone subunit beta)	(184, 206)
Ruminococcus	Firmicutes	Positive correlations with LPS, TNF and TRACP-5b	(205)
Burkholderiales	Betaproteobacteria	Strong positive correlations with Tb.N, ALP, claudin-1, and occludin; repairing the intestinal barrier; increased BMD;elimination of potentially pathogenic cytokines	(205)
Parabacteroides	Bacteroidetes	Positive regulation in glucose and lipid metabolism.;strong positive correlations with Tb.BMD and Tb.N	(205)
Alistipes	Bacteroidetes	Strong positive correlations with Intestinal barrier markers	(205)
Parasutterella	Betaproteobacteria	Strong positive correlations with ALP and BGP	(205)
Muribaculum	Bacteroidetes	Regulation of T cells; exhibited strong negative correlations with intestinal inflammatory factors and bone resorption factors; strong positive correlations with intestinal barrier proteins and bone formation factors.	(205)
Lactobacillus plantarum	Firmicutes	Dysbiosis correction	(207)
Bacillus clausii	Firmicutes	Regulation of Th17/Treg immune homeostasis	(217)
Faecalibacterium prausnitzii	Firmicutes	Osteogenic differentiation suppression via butyrate production	(220)

RA, rheumatoid arthritis; AKT2, serine/Threonine Kinase 2; EVs, extracellular vesicles; LPS, Lipopolysaccharide; TNF, Tumor necrosis factor; TRACP, tartrate resistant acid phosphatase; Tb.N, trabecular number; ALP, alkaline phosphatase; BMD, bone mineral density; BGP, bone-γ-Carboxyglutamic Acid-Containing protein; SAA, serum amyloid A; TGF-β, transforming growth factor-β; IL-10, interleukin 10; DCs, dendritic cells; NOD2, nucleotide-binding oligomerization domain 2; GIP, glucose - dependent insulinotropic polypeptide; PYY, peptide YY.

A metagenomic study conducted in both Chinese and American populations revealed that *Bacteroides vulgatus* (*B. vulgatus*) was negatively correlated with BMD, while the metabolite serum valeric acid (VA) showed a positive correlation with BMD. Subsequent animal experiments confirmed that VA suppresses the production of the pro-inflammatory factor RELA and enhances the mRNA expression of the anti-inflammatory cytokine IL-10, thereby inhibiting bone resorption. In contrast, *B. vulgatus* was found to promote bone resorption by inhibiting VA production, highlighting the potential of the *Bacteroides*-valeric acid axis as a therapeutic target (204). A Mendelian randomization analysis further demonstrated that the abundance of *Burkholderiales* was strongly positively correlated with bone formation markers, gut barrier indicators (e.g., claudin-1, Claudin), and bone density parameters (e.g., Tb.N, ALP). Conversely, the *genus Ruminococcus* exhibited strong positive correlations with bone resorption markers and gut inflammatory factors (e.g., LPS, TNF, TRACP-5b). The study also identified *Akkermansia*, *Parabacteroides*, *Alistipes*, *Parasutterella*, and *Muribaculum* as potential mediators for the clinical diagnosis and treatment of OP (205). Additionally, metabolomic investigations revealed that *L. reuteri* promotes the production of hormones such as serotonin, GIP, and PYY, including vasopressin and luteinizing hormone subunit beta—newly identified hormones produced in gut epithelial cells—thereby broadening our understanding of microbial regulatory mechanisms in bone metabolism (206).

In addition to predicting potential therapeutic targets and biomarkers for OP, omics approaches have been utilized to validate the efficacy of pharmacological treatments, providing robust experimental evidence and offering new directions for elucidating microbial mechanisms. For instance, studies have demonstrated that *Lactobacillus plantarum* and curcumin increase beneficial GM while reducing harmful species, thereby ameliorating glucocorticoid-induced OP (207, 208). Another study revealed that alginate oligosaccharides alleviate estrogen-deficient osteosarcopenia by modulating bile acid metabolism, reducing Th17 cell prevalence and systemic inflammation (209). Additionally, a Chinese herbal extract, Isaria felina, was found to improve bone metabolism by correcting gut dysbiosis (particularly in *Bacteroides* and *Ruminococcus*), restoring Th17/Treg balance, and lowering levels of inflammatory cytokines such as IL-17 and TNF-α. Metabolomic analyses further indicated alterations in nucleotide and lipid metabolism (210).

The rational application of these advanced biotechnologies will facilitate the discovery of novel biomarkers and therapeutic targets, paving the way for the development of safer and more effective interventions.

## 6 How does gut flora affect bone metabolism through the immune system

**Figure 2:**
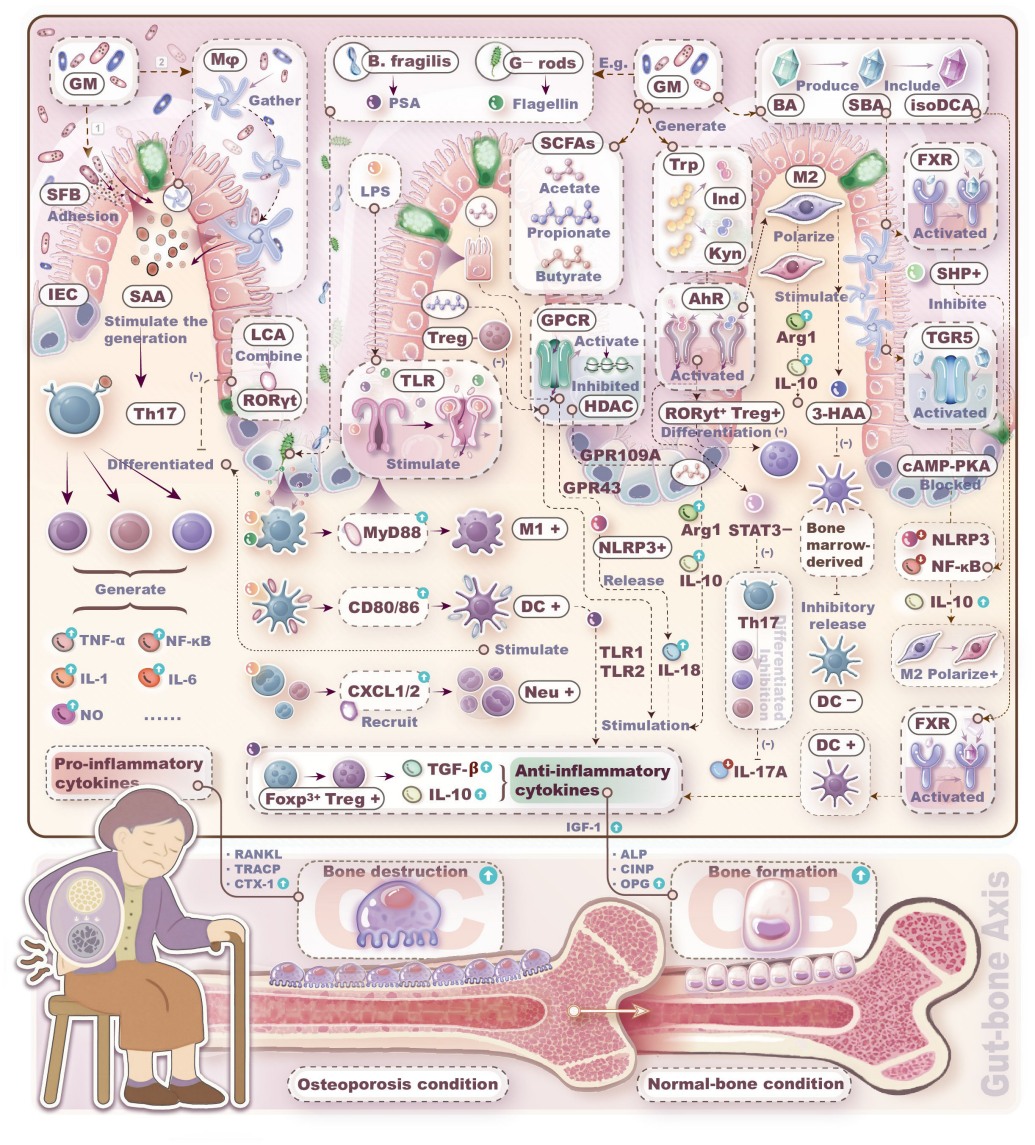
Mechanisms by which gut microbiota regulates bone metabolism through immune cells. Bacteria themselves, bacterial components, and bacteria metabolites can induce immune cells to produce pro-/anti-inflammatory factors, thereby promoting either bone resorption or bone formation.

Through comprehensive analysis of the GM-immune cell-OP relationship, we have established that the Th17/Treg equilibrium, Breg cells, and macrophage polarization profiles play key roles in maintaining bone homeostasis. As gut microbes continuously stimulate these immune cells, the GM thus indirectly influences bone metabolism via the immune system. The crosstalk mechanisms within the GM-immune cells-OP axis is described in **Figure 2**. Although mechanistic links between GM and skeletal health involve profound complexity, current hypotheses remain overwhelmingly focused on T-lymphocyte-mediated pathways. Research on non-T immune cells is critically underexplored. Beyond this T cell-centric paradigm, emerging evidence reveals that macrophages and DCs constitute a pivotal regulatory triumvirate with B lymphocytes. Neutrophils, NK, and NKT cells likewise function as putative modifiers of microbe-directed osteometabolic regulation.

While the mechanisms linking GM to skeletal homeostasis remain elusive, current hypotheses derived from recent literature have predominantly focused on T lymphocytes. Besides the mainstream proposed T-cell-mediated interactions, we have found that macrophages and dendritic cells may play pivotal roles, while B lymphocytes, neutrophils, NK cells, and NKT cells could also serve as potential targets in microbiota-mediated regulation of bone metabolism.

As delineated, bacterial flagellin activates B-lymphocyte proliferation/differentiation, triggering IgA-mediated pathogen neutralization. LPS polarizes macrophages toward pro-osteolytic M1 phenotypes that secrete bone-resorptive cytokines; conversely, microbial metabolites (e.g., SCFAs, tryptophan derivatives, secondary bile acids) induce anti-inflammatory M2 polarization while suppressing Th17 expansion. LPS and flagellin provoke DC inflammatory cascades via TLR/MyD88 pathways, whereas microbial metabolites (butyrate, kynurenine, isoDCA) attenuate DC immunogenicity by inducing IL-10 and Treg differentiation. Additionally, LPS releases CXCL1/2 to recruit neutrophils and primes NK cytolytic function. Thus, current limited evidence unequivocally underscores an urgent need for comprehensive investigations into multicellular crosstalk mechanisms orchestrating immune functions within the gut-bone axis. Elucidating gut-immunological pathways and decoding dysbiosis-induced pathophysiological cascades may unlock mechanistic insights into osteometabolic disorders.

Furthermore, microbial metabolites act directly on IECs, modulating gut barrier integrity and functionality—thereby indirectly governing microbiota-immune interplay. Compromised epithelial barrier integrity precipitates pathogen translocation into systemic circulation, triggering sterile inflammation and potentiating gastrointestinal pathologies (211). Critically, gut barrier dysfunction accelerates bone mass deterioration as demonstrated in preclinical models (212). A recent study analyzing fecal samples from postmenopausal women revealed a significant reduction in the abundance of *Prevotella* compared to healthy controls. Subsequent animal experiments demonstrated that treatment with *Prevotella histicola* partially restored bone mass in OVX mice, a effect potentially mediated through the modulation of intestinal permeability (213). Another study indicated that overexpression of neuropeptide Y exacerbates colonic inflammation and impairs gut barrier integrity in OVX rats, thereby increasing the systemic translocation of GM-derived metabolites such as LPS. These adverse effects were reversed following administration of a Y1 receptor antagonist (214). Furthermore, in a diabetic mouse model, treatment with angiotensin- (1-7) increased the abundance of *Firmicutes*, promoted the restoration of intestinal stem cells, and ameliorated diabetes-induced impairment of colonic barrier function (215). Collectively, these findings underscore the importance of intestinal permeability as a critical factor in elucidating the regulatory mechanisms of the gut-bone axis.

## 7 Intervention strategies based on the microbiome-immune-bone axis

**Figure 3:**
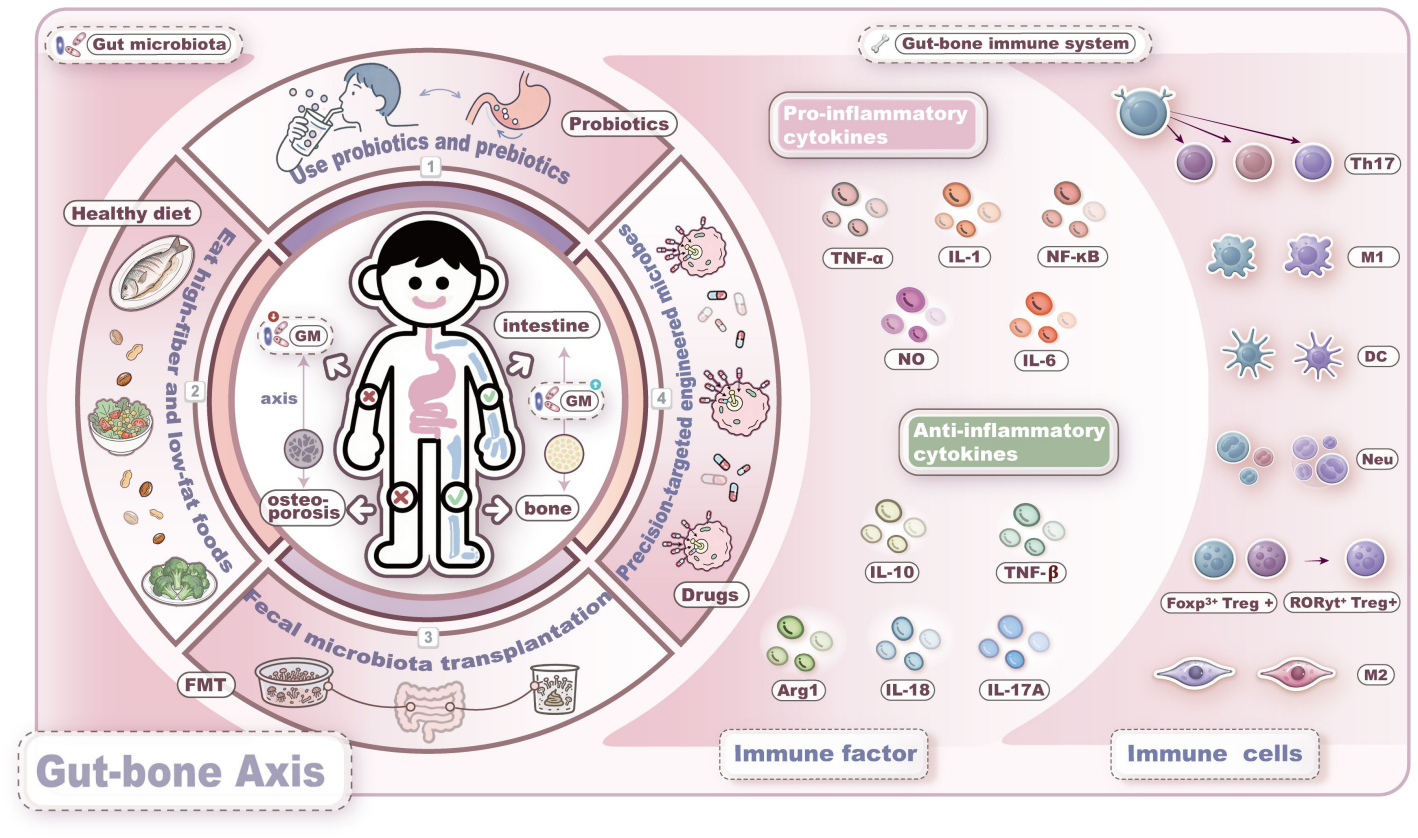
Intervention strategies based on the microbiome-immune-bone axis. Current strategies for gut microbiota-targeted therapy mainly include the following four approaches: 1. probiotics and prebiotics; 2. dietary interventions; 3. fecal microbiota transplantation; 4. engineered microbes. These strategies aim to improve bone metabolism by restoring gut microbiota dysbiosis, modulating immune responses, enhancing the gut barrier function, and regulating the endocrine system.

Conventional anti-OP drugs are primarily classified into two categories: anti-resorptive and anabolic agents. However, their clinical utility is limited by adverse effects. In contrast, emerging microbiota-targeted therapies offer unique advantages, which are summarized in **Figure 3**.

### 7.1 Probiotics, prebiotics and diet

In the second section, we summarize the mechanisms by which various probiotics and prebiotics ameliorate OP, including reduction of inflammatory factors, alteration of metabolites, regulation of immune and endocrine systems, and enhancement of the intestinal barrier. Animal studies have demonstrated that *Lactobacillus acidophilus*, *Bacillus clausii*, and *Lactobacillus rhamnosus* alleviate inflammatory bone loss in osteoporotic mouse models by modulating Treg/Th17 cell balance, highlighting the therapeutic potential of the microbiota-immune axis (216–218). Additionally, the indole-derived metabolite melatonin significantly increases SCFAs production and reduces trimethylamine N-oxide (TMAO)-related metabolites. By restoring M1/M2 macrophage equilibrium and lowering pro-inflammatory cytokine levels, MLT ultimately attenuates OP-related clinical symptoms and reverses gut dysbiosis, supporting the microbial-metabolite-immune network as a promising therapeutic target (219). Notably, butyrate produced by *Faecalibacterium prausnitzii* competitively inhibits lactylation at lysine 263 of GAPDH via induction of butyrylation at the same site, thereby suppressing the osteogenic differentiation of human aortic valve interstitial cells. This mechanism reveals the potential of butyrate in treating calcific aortic valve disease and offers novel insights into metabolite-mediated regulation of osteogenesis (220). Further evidence indicates that *L. reuteri* prevents bone loss by reversing antibiotic-induced gut dysbiosis and restoring intestinal barrier integrity, underscoring the importance of microbiota-modulated gut permeability in skeletal health (221). Moreover, synbiotics—formulations combining probiotics and prebiotics—modulate multiple gut microbial metabolites and improve bone metabolic markers, positioning them as promising next-generation interventions for OP (222).

Dietary patterns exert a decisive influence on the composition, diversity, and function of the GM (126). Experimental studies have demonstrated that a high-fiber diet restores Th17/Treg balance, enhances intestinal barrier integrity, and increases the abundance of SCFAs, thereby ameliorating autoimmune pathology in RA and significantly reducing bone loss (223, 224). A randomized controlled trial (RCT) revealed that blackcurrant supplementation increased the relative abundance of *Ruminococcus 2* and effectively improved BMD in patients with PMOP (225). Another large three-year RCT involving 924 elderly participants demonstrated that a low-calorie Mediterranean diet intervention effectively mitigated age-related decline in BMD, confirming the efficacy of dietary therapy in ameliorating bone loss (226). Therefore, dietary strategies should be incorporated into clinical practice as foundational adjuvant treatments to enhance therapeutic outcomes in OP patients.

### 7.2 Fecal microbiota transplantation

Fecal microbiota transplantation (FMT) is a therapeutic approach that involves extracting and transplanting beneficial microbial communities from healthy donor feces into the gastrointestinal tract of recipients to remodel gut microbial composition (227). The efficacy of FMT in treating OP primarily operates through improved intestinal barrier function, correction of GM dysbiosis, and modulation of immune responses to regulate bone metabolism (228). Animal studies have demonstrated that FMT upregulates tight junction proteins—such as zonula occludens-1 (ZO-1) and occludin—and inhibits the release of pro-osteoclastic cytokines, including TNF-α and IL-1β. It also optimizes the composition and abundance of GM, concomitantly elevating levels of acetate and propionate (229). Another study revealed that combining exercise with FMT enhances the enrichment of bile acid metabolites, including taurocholic acid and ursodeoxycholic acid, thereby mediating protective effects on bone mass (230).

Currently, the application of FMT in anti-OP therapy remains in the experimental stage, with limited clinical data available. The lack of standardized treatment protocols—including administration routes, donor selection, microbiota preparation, and storage methods—may influence treatment outcomes (227). Furthermore, FMT entails safety concerns such as the transmission of infectious diseases, adverse immune reactions, and long-term complications related to dysbiosis. Its therapeutic effects are often transient, necessitating repeated transplants or adjunct interventions to sustain microbial homeostasis, which presents ongoing challenges for clinical standardization. Future clinical studies should adhere to high safety standards, extend follow-up periods beyond one year to monitor potential long-term risks, and establish unified and standardized procedural guidelines.

### 7.3 Precision targeting therapies of engineered microbes

The engineering of probiotics using synthetic biology for precision-targeted therapy has emerged as a representative next-generation biotherapeutic strategy (231). A biomimetic self-adjuvating vaccine developed from programmable macrophage-derived vesicles significantly enhanced the activation rate of antigen-presenting cells by nearly fourfold at equivalent dosages against monkeypox virus, demonstrating substantial potential of engineered biological systems (232). Through a co-culture system, intestinal organoids (IOs) and their EVs were applied in combination, resulting in enhanced intestinal barrier integrity, reduced expression of inflammatory factors, and effective amelioration of inflammatory bowel disease-associated OP. This finding highlights the clinical translational potential of IOs and their EVs (233). Another research team improved microbial-based delivery methods by developing a colon-targeted drug delivery system using shellac resin-coated polyvinyl butyral nanoparticles, enabling sustained release of the postbiotic butyrate in the colorectum. This system effectively suppressed macrophage-mediated inflammation and modulated GM composition, highlighting its promise for targeted immuno-microbial therapy (234). In OVX mice, outer membrane vesicles (OMVs) derived from *Proteus mirabilis* inhibited osteoclastogenesis by elevating ROS and inducing mitochondrial dysfunction, thereby mitigating experimental bone loss (235). Researchers also constructed a recombinant probiotic *Escherichia coli* strain and isolated engineered BEV-BMP-2-CXCR4 vesicles (BEVs-BC), which markedly promoted osteogenic differentiation of BMSCs (236). Additionally, engineered small EVs modified with EXOmotif (CGGGAGC) and loaded with anti-miR-6359 exhibited precise targeting toward OCPs and ameliorated valproic acid-induced bone loss (237).

Collectively, these studies underscore the encouraging progress in employing engineered microbes to restore bone metabolic homeostasis, though clinical translation will require the establishment of stringent standardized protocols.

### 7.4 Clinical translation

Intervention strategies based on the microbiota-immune-bone axis have emerged as a promising approach for the clinical management of OP. A RCT demonstrated that supplementation with *L. reuteri* effectively prevented the deterioration of GM and inflammatory status in elderly women with low BMD (238). However, another double-blind, randomized, placebo-controlled clinical trial using *L. reuteri* did not yield positive outcomes, potentially due to insufficient dosage or viability of the probiotic strain (239). Combined supplementation with *Bifidobacterium lactis* subsp. Probio-M8, calcium, and calcitriol proved more effective than conventional drug therapy alone, providing a theoretical foundation for synergistic probiotic/prebiotic-mineral matrix combination strategies (240). Traditional Chinese medicine has also shown potential in modulating the gut-bone axis; clinical trials revealed that integrative therapy with conventional treatment and Chinese herbal medicine improved GM composition and modulated metabolite profiles—such as diclofenac, carbamazepine, D-pyroglutamic acid, and tamsulosin—facilitating recovery in OP patients (241).

Despite these promising findings, existing clinical trials are limited by small sample sizes, short follow-up durations, and considerable microbial heterogeneity. Safety concerns regarding GM interventions also warrant careful consideration, as complications from FMT may lead to unpredictable outcomes. Moreover, probiotics often influence bone metabolism through multiple pathways and targets, and systemic administration frequently results in low bioavailability at the intended sites and off-target effects. Therefore, achieving precise microbial intervention remains a significant challenge for clinical translation. Future advances in nanodelivery systems for engineered microbes may provide a viable solution.

## 8 Conclusions and future directions

The GM regulates bone metabolism through multiple mechanisms, including modulation of inflammatory factors, alterations in metabolites, and interactions with the immune and endocrine systems, as well as maintenance of intestinal barrier integrity. This review focuses on the interactions between GM and immune cells, and between immune cells and OP, aiming to elucidate and establish a comprehensive GM-immune cell-OP crosstalk network. Intervention strategies based on the microbiota-immune-bone axis show therapeutic potential in OP management, with promising applications of probiotics, prebiotics, dietary patterns, FMT, and engineered microbes. Nevertheless, numerous challenges remain unresolved, and future efforts should prioritize the following aspects.

Although preliminary insights into the GM-immune-bone axis have been established, the precise regulatory mechanisms remain to be fully elucidated. The considerable heterogeneity of GM and their metabolites, coupled with their multi-pathway, multi-receptor, and multi-target modes of action, make it exceptionally challenging to decipher their exact mechanisms. Future mechanistic studies should prioritize understanding how specific microbial species influence bone metabolism through distinct immune cells. Microbiome research may hold the key to accelerating progress in this area. Although multi-omics approaches—as discussed in Section 5—have helped correlate microbial and immune signatures with bone metabolic markers, the underlying mechanisms remain elusive. Future investigations should employ integrated biological animal models, combining advanced transcriptomic, proteomic, and multi-omics technologies to systematically unravel the mechanisms of microbial communities and their metabolites, thereby facilitating the development of robust biomarkers for precision medicine.

On the other hand, a substantial body of preclinical evidence supports the role of the GM in indirectly improving skeletal health through immune cell mediators, corroborating the mechanisms discussed herein. However, translating these foundational insights into effective and clinically viable applications remains a major challenge. In contrast, clinical studies in this area remain scarce and have produced inconsistent outcomes, particularly due to a lack of high-quality trials. Interindividual variability and microbial heterogeneity likely contribute to these discrepancies. For instance, to address compositional variability in the microbiome, future approaches could employ personalized bacterial profiling to select appropriate microbial consortia for precision therapeutics aimed at restoring skeletal health. Equally critical is the design of microbial interventions—such as FMT and engineered microbes—that can safely, precisely, and effectively target the immune system to maintain bone metabolic homeostasis. There is an urgent need for large-scale, multicenter RCTs with long-term follow-up, which must incorporate appropriate patient stratification based on complex microbial or metabolic signatures to rigorously evaluate the impact of microbiota-targeted therapies on key clinical outcomes and enhance the scientific rigor and translational credibility of the evidence.
